# Fluctuating NMDA Receptor Subunit Levels in Perirhinal Cortex Relate to Their Dynamic Roles in Object Memory Destabilization and Reconsolidation

**DOI:** 10.3390/ijms22010067

**Published:** 2020-12-23

**Authors:** Cassidy E. Wideman, James Nguyen, Sean D. Jeffries, Boyer D. Winters

**Affiliations:** Department of Psychology and Collaborative Neuroscience Program, University of Guelph, Guelph, ON N1G 2W1, Canada; jnguye18@uoguelph.ca (J.N.); sjeffrie@uoguelph.ca (S.D.J.); bwinters@uoguelph.ca (B.D.W.)

**Keywords:** memory, destabilization, reconsolidation, glutamate, acetylcholine, boundary conditions

## Abstract

Reminder cues can destabilize consolidated memories, rendering them modifiable before they return to a stable state through the process of reconsolidation. Older and stronger memories resist this process and require the presentation of reminders along with salient novel information in order to destabilize. Previously, we demonstrated in rats that novelty-induced object memory destabilization requires acetylcholine (ACh) activity at M_1_ muscarinic receptors. Other research predominantly has focused on glutamate, which modulates fear memory destabilization and reconsolidation through GluN2B- and GluN2A-containing NMDARs, respectively. In the current study, we demonstrate the same dissociable roles of GluN2B- and N2A-containing NMDARs in perirhinal cortex (PRh) for object memory destabilization and reconsolidation when boundary conditions are absent. However, neither GluN2 receptor subtype was required for novelty-induced destabilization of remote, resistant memories. Furthermore, GluN2B and GluN2A subunit proteins were upregulated selectively in PRh 24 h after learning, but returned to baseline by 48 h, suggesting that NMDARs, unlike muscarinic receptors, have only a temporary role in object memory destabilization. Indeed, activation of M_1_ receptors in PRh at the time of reactivation effectively destabilized remote memories despite inhibition of GluN2B-containing NMDARs. These findings suggest that cholinergic activity at M_1_ receptors overrides boundary conditions to destabilize resistant memories when other established mechanisms are insufficient.

## 1. Introduction

Initially, memories exist in an active, labile state before stabilizing for storage in long-term memory via protein-synthesis-dependent consolidation [1,2]. The nature of long-term memory storage is more dynamic than once believed, such that the presentation of reminder cues can destabilize consolidated memories, rendering them labile and vulnerable to modification [3,4] before undergoing a second protein synthesis dependent re-stabilization referred to as reconsolidation [3,5]. Memory destabilization and subsequent reconsolidation is widely believed to represent a neurobiological process by which memories can be maintained accurately over time [6,7].

Several neuromodulators of memory destabilization and reconsolidation have been identified in recent years [8]. Glutamate was first implicated in reconsolidation for a number of memory systems in different brain regions, and it has been demonstrated that inhibiting activity at N-methyl-D-aspartate (NMDA) receptors or NMDRs prevents reconsolidation [5,9,10,11,12,13,14,15,16]. However, it was also shown that antagonizing NMDARs in the basolateral amygdala (BLA) prior to fear memory reactivation prevented memory disruption by post-reactivation protein synthesis inhibition, suggesting that these receptors also have a role in memory destabilization [17].

Typically, NMDARs exist in the central nervous system as tetramers composed of glycine-binding GluN1, glutamate-binding GluN2, and glycine-binding GluN3 subunits [18,19]. The GluN2 subunit has four subtypes (GluN2A-D), each with distinct functional properties that can diversify NMDAR kinetics based on subunit composition [19]. A study using antagonists selective to different GluN2 receptor subtypes revealed dissociable involvement of GluN2B- and GluN2A-containing NMDARs in fear memory destabilization and reconsolidation, respectively [15]. This provided an explanation for how glutamate can regulate both processes in a manner that is dependent on receptor subunit composition [15]. It was hypothesized that activation of GluN2B-containing NMDARs may lead to downstream activation of the ubiquitin proteasome system (UPS), an important regulator of protein dynamics in the post-synaptic density [15,20,21,22]. Indeed, UPS-mediated synaptic protein degradation is thought to provide a physiological correlate for memory destabilization at the synaptic level [21,22,23,24,25].

However, not all memories destabilize following reactivation. Memories that are strongly encoded or remote at the time of reactivation resist destabilization, suggesting that there are boundary conditions on this process [16,26,27,28,29,30]. Studies using fear conditioning demonstrate that strongly encoded fear memories that are resistant to destabilization are associated with a reduction in GluN2B-containing NMDARs in the BLA [31]; that is, as the number of tone–foot shock pairings increase to strengthen the memory, there is an increase in the ratio of GluN2A to GluN2B-containing NMDARs [31,32]. Similarly, preventing the downregulation of GluN2B-containing NMDARs in the BLA abolishes resistance to destabilization [32]. These findings provide strong evidence for a mechanism that could explain boundary conditions on memory destabilization, whereby receptors required to induce destabilization are downregulated, thereby preventing initiation of the entire process [33].

These boundaries on memory lability may represent an adaptive mechanism for protecting remote and strongly encoded memories, such that they resist destabilization and subsequent modification. Another theory suggests that there must be sufficient mismatch or prediction error between the reactivation episode and initial learning conditions for certain memories to destabilize [33,34]. Consistent with this idea, we have previously demonstrated that resistant object memories can be destabilized when novel contextual information is presented along with reminder cues at the time of reactivation [16]. The presentation of new information at the time of reactivation may signal the opportunity for updating the memory trace, and thus promote destabilization to allow for the incorporation of new information. We have shown that both standard destabilization (in the absence of boundary conditions) and novelty-induced destabilization of resistant object memories are dependent on acetylcholine (ACh) activity at M_1_ muscarinic receptors (mAChR) in perirhinal cortex (PRh) and provided evidence for the cellular pathway by which M_1_ receptors promote destabilization through downstream activation of the UPS [24,35].

Thus, there is evidence that both M_1_ mAChR and GluN2B-containing NMDAR activation at the time of memory reactivation lead to increased intracellular calcium and the recruitment of the UPS to promote memory destabilization [32,33]. The purpose of the current study was to begin to bring together research regarding the roles of ACh and glutamate in destabilizing both readily destabilized and resistant memories in order to determine their combined or unique contributions to this memory maintenance process. We hypothesized that GluN2B- and GluN2A-containing NMDARs would be involved in destabilizing and reconsolidating relatively recent object memories in PRh, respectively; however, GluN2B-containing NMDARs would be downregulated for older object memories that resist destabilization, negating their involvement in novelty-induced object memory destabilization. The results of this study, which support our main predictions, suggest that NMDARs in PRh have only a temporary role in object memory destabilization; this contrasts with the function of ACh, which appears to serve an important, NMDAR-independent role in circumventing boundary conditions to destabilize resistant memories.

## 2. Results

### 2.1. Histology

**Figure 1:**
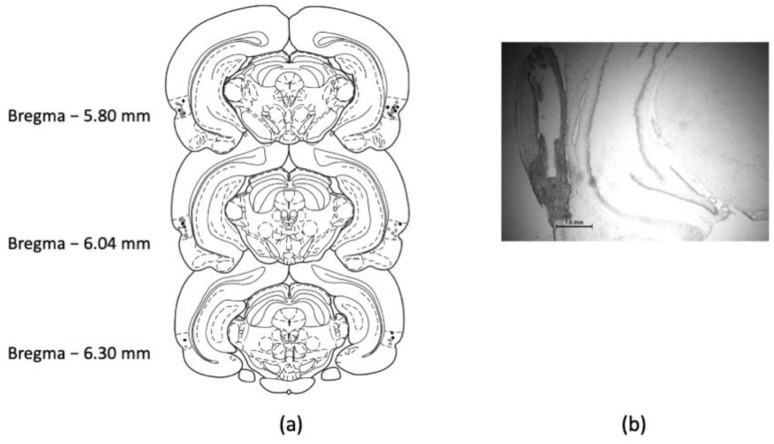
Cannula placements in perirhinal cortex (PRh): (**a**) schematic of infusion tip placements from all rats used in behavioral experiments; (**b**) micrograph showing guide cannula tract with the infusion tip terminating near the rhinal sulcus.

All rats included in final behavioral analyses had bilateral guide cannulae with infusion needle tips terminating in PRh. Infusion tips were located at the border between areas 35 and 36, represented by the rhinal sulcus [36]. Placements were consistently located between 5.80 and 6.30 mm posterior to bregma (Figure 1).

### 2.2. GluN2B-Containing NMDARs Are Required in PRh for Destabilization, but Not Reconsolidation, of Relatively Recent Object Memories

**Figure 2:**
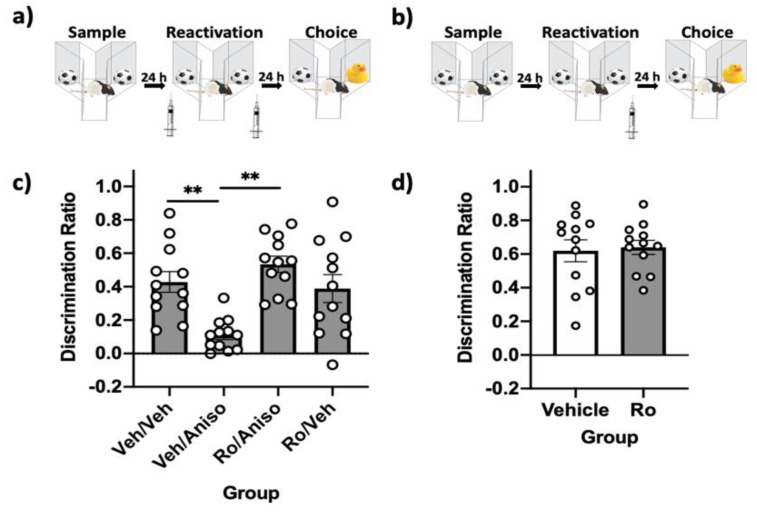
GluN2B-containing NMDARs in PRh are involved in destabilizing, but not reconsolidating, relatively recent object memories. (**a**) Standard SOR parameters, with immediate pre-reactivation PRh infusions to target destabilization and prevent the impairing effects of post-reactivation infusions of the reconsolidation inhibitor anisomycin. (**b**) Standard SOR parameters, with only post-reactivation intra-PRh infusions to target memory reconsolidation. (**c**) Choice-phase DRs. All groups’ choice DRs differed significantly from their respective sample DR, with the exception of the veh–aniso group, suggesting memory was impaired in this group. The veh–veh and Ro–aniso groups had significantly better memory performance during the choice phase compared to the veh–aniso group. (**d**) Choice-phase DRs. Both groups’ choice DRs differed significantly from their respective sample DRs, suggesting memory was intact, while performance during the choice phase did not differ between groups. Bars are mean DR ± standard error of the mean (SEM); ** *p* < 0.01.

These experiments investigated the involvement of GluN2B-containing NMDARs in destabilizing and reconsolidating relatively recent (24 h) object memories in PRh. First, the “standard” spontaneous object recognition (SOR) parameters were used to assess the role of these receptors in object memory destabilization, with rats (*n* = 12) receiving a pre-reactivation infusion of vehicle or the selective GluN2B-containing NMDAR antagonist Ro 25-6981 (Ro) and a post-reactivation infusion of vehicle or anisomycin (Figure 2a). Immediate post-reactivation infusions of the reconsolidation inhibitor anisomycin impaired object memory. However, pre-reactivation infusion of Ro prevented this impairment (Figure 2c). A repeated measures analysis of variance (ANOVA) comparing choice discrimination ratios (DRs) demonstrated a significant effect of the drug (*F*(3,33) = 8.82, *p* = 0.00, *η*^2^ = 0.45). Post-hoc tests demonstrated that memory in the vehicle–anisomycin condition was significantly impaired relative to the vehicle–vehicle condition (*p* = 0.007). A paired-sample *t*-test comparing the sample-to-choice DR was not significantly different for the vehicle–anisomycin condition (*t*(11) = −0.315, *p* = 0.78), suggesting absence of recognition in this condition; all other conditions were significantly different (all *p* values < 0.01).

A repeated measures one-way ANOVA revealed a significant difference in total object exploration between groups during the sample phase (*F*(3,33) = 17.99, *p* = 0.00, *η*^2^ = 0.62). Post-hoc test demonstrated that there was significantly lower exploration in the Ro–Veh condition compared to the Ro–anisomycin (*p* = 0.004) and the vehicle–anisomycin condition (*p* = 0.00); however, this group displayed intact object memory during the choice phase suggesting this reduction in exploration did not affect memory performance. Similarly, there was reduced exploration in the vehicle–vehicle condition compared to the vehicle–anisomycin condition (*p* = 0.004), but memory was intact in the choice phase, suggesting the reduced exploration did not negatively affect memory.

Next, using the same standard SOR parameters, we determined that immediate post-reactivation infusion of Ro had no effect on memory performance (Figure 2b). This suggests that GluN2B-containing NMDARs are not involved in reconsolidating relatively recent object memories (Figure 2d). A paired-sample *t*-test demonstrated that choice DRs were not significantly different (*t*(11) = 0.24, *p* = 0.82) when rats were given vehicle compared to Ro following reactivation. Both the vehicle and Ro condition displayed intact memory during the choice phase, as paired-sample *t*-tests comparing sample-to-choice DRs were significantly different in both conditions (all *p* values < 0.001).

### 2.3. GluN2A-Containing NMDARs Are Required in PRh for Reconsolidation, but Not Destabilization, of Relatively Recent Object Memories

**Figure 3:**
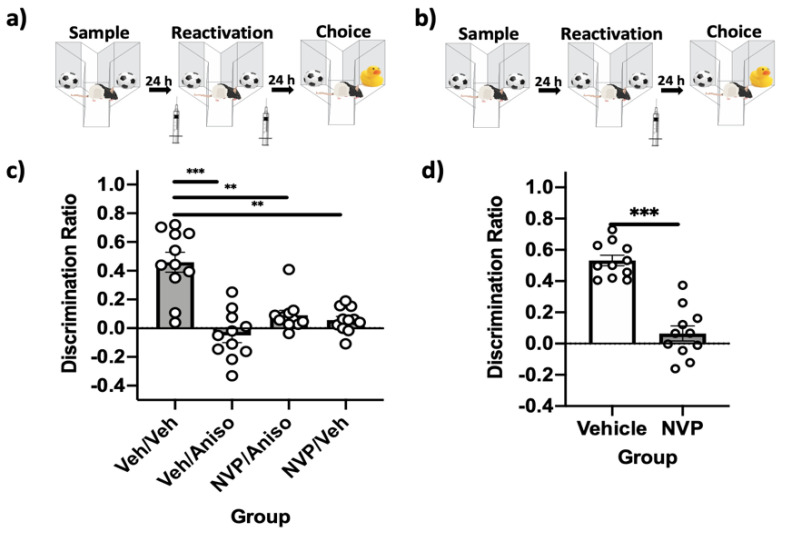
GluN2A-containing NMDARs in PRh are involved in reconsolidating, but not destabilizing, relatively recent object memories. (**a**) Standard SOR parameters, with intra-PRh infusions performed immediately prior to reactivation to target destabilization and prevent the impairing effects of post-reactivation infusion of anisomycin. (**b**) Standard SOR parameters, where only post-reactivation intra-PRh infusions were performed to target reconsolidation. (**c**) Choice-phase DRs. Only the veh–veh group had a choice DR that differed from its respective sample DR, suggesting this was the only group with intact memory. This group had significantly greater performance than all other groups. (**d**) Choice-phase DRs. The NVP group choice DR did not differ from its respective sample DR, suggesting lack of recognition memory, while memory was significantly impaired compared to the veh group. Bars are mean DR ± SEM; ** *p* < 0.01, *** *p* < 0.001.

These experiments investigated the involvement of GluN2A-containing NMDARs in PRh in destabilizing and reconsolidating relatively recent (24 h) object memories (*n* = 11). Using the standard SOR parameters (Figure 3a), immediate post-reactivation infusion of the protein synthesis inhibitor anisomycin impaired object memory. This impairment persisted even when the selective GluN2A-containing NMDA receptor inhibitor NVP-AAM 077 (NVP) was infused prior to reactivation, suggesting that these receptors are not required for destabilization when object memories are relatively recent (Figure 3c). A repeated measures one-way ANOVA revealed a significant effect of the drug (*F*(3,30) = 21.88, *p* = 0.00, *η*^2^ = 0.69) on choice DR. Post-hoc analysis determined that performance in the vehicle–anisomycin, NVP–anisomycin, and NVP–vehicle conditions were significantly impaired relative to the vehicle–vehicle condition (all *p* values < 0.01). Paired-sample *t*-tests revealed that sample and choice DRs were not significantly different in the vehicle–anisomycin, NVP–anisomycin, and NVP–vehicle conditions (all *p* values > 0.05), suggesting an absence of object recognition memory. Memory was intact in the vehicle–vehicle condition, as demonstrated by a significant difference between sample and choice DR (*t*(10) = −6.38, *p* = 0.00).

A repeated measures one-way ANOVA demonstrated a significant effect of the drug on total exploration (*F*(3,30) = 4.22, *p* = 0.01, *η*^2^ = 0.3) during the reactivation phase, but post-hoc analysis revealed no significant differences between the four groups.

The standard SOR parameters were then used to assess the role of GluN2A-containing NMDARs in PRh in reconsolidating relatively recent (24 h) object memories (Figure 3b). Immediate post-reactivation infusion of NVP impaired memory performance (Figure 3d). A paired samples *t*-test demonstrated that choice DRs were significantly different (*t*(10) = 10.96, *p* = 0.00) when rats were given vehicle infusions compared to NVP infusions following reactivation. Paired-sample *t*-tests comparing sample-to-choice DRs demonstrated that memory was intact in the vehicle condition (*t*(10) = −11.16, *p* = 0.00) and memory was impaired in the NVP condition (*t*(10) = −0.73, *p* = 0.49).

The vehicle condition had significantly reduced object exploration during the sample (*t*(10) = −3.98, *p* = 0.003) and choice phases (*t*(10) = −2.83, *p* = 0.02). This reduced exploration did not appear to affect performance, as object recognition memory was intact during the choice phase.

### 2.4. GluN2B-Containing NMDARs in PRh Are Not Required for Destabilizing or Reconsolidating Remote Object Memories

**Figure 4:**
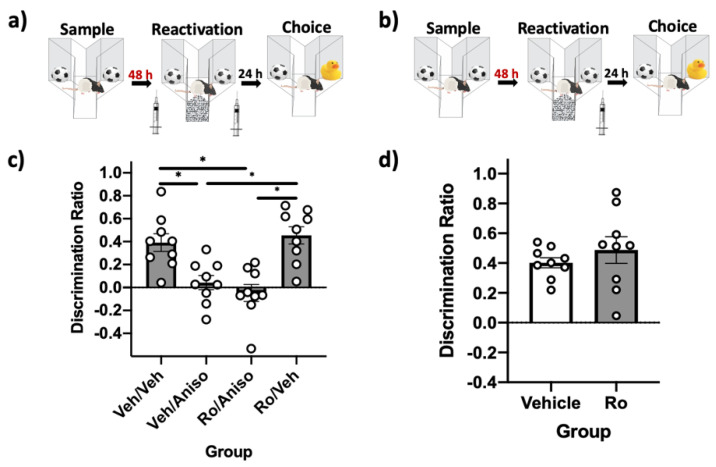
GluN2B-containing NMDARs in PRh are not involved in destabilizing or reconsolidating remote (48h) object memories. (**a**) Remote SOR parameters, where contextual novelty in the form of a floor insert is required at the time of reactivation to destabilize remote object memories. PRh infusions were performed immediately prior to reactivation to target destabilization and prevent the impairing effects of post-reactivation infusion of anisomycin. (**b**) Remote SOR parameters, where only post-reactivation PRh infusions were performed to target reconsolidation. (**c**) Choice-phase DRs. The veh–veh and Ro–veh groups had choice DRs that differed from their respective sample DRs, suggesting that these groups had intact memory. Memory in both groups receiving post-reactivation anisomycin was significantly impaired compared to vehicle conditions. (**d**) Choice-phase DRs, demonstrating no differences in memory performance. Both groups’ choice DRs differed significantly from their respective sample DRs, suggesting memory was intact. Bars are mean DR ± SEM; * p < 0.05.

These experiments investigated the role of GluN2B-containinig NMDARs in PRh for destabilizing (*n* = 9) and reconsolidating (*n* = 9) remote object memories. Using the “remote” SOR parameters (48 h) with an insert placed on the floor of the apparatus to serve as contextual novelty during reactivation (Figure 4a), immediate post-reactivation infusion of the protein synthesis inhibitor anisomycin impaired object memory, indicating that these conditions are sufficient to destabilize remote object memories, as previously demonstrated [16]. Unlike findings using the standard SOR parameters, pre-reactivation infusion of the selective GluN2B-containing NMDAR antagonist Ro did not prevent this impairment (Figure 4c). A repeated measures ANOVA comparing DRs during the choice phase revealed a significant effect of the drug (*F*(3,24) = 10.98, *p* = 0.00, *η*^2^ = 0.58). Post-hoc tests demonstrated that performance in the vehicle–anisomycin (*p* = 0.02) and the Ro–anisomycin (*p* = 0.05) condition were significantly impaired relative to vehicle–vehicle. Paired-sample *t*-tests comparing sample-to-choice DR were not significantly different for the vehicle–anisomycin (*t(*8) = −0.03, *p* = 0.98) and the Ro–anisomycin conditions (*t*(8) = 0.44, *p* = 0.68), suggesting absence of recognition in these conditions; all other conditions were significantly different (all *p* values < 0.01).

A repeated measures one-way ANOVA revealed a significant effect of the drug on total exploration during the reactivation phase (*F*(3,24) = 6.87, *p* = 0.002, *η*^2^ = 0.46), but post-hoc tests indicated no significant differences in exploration between groups.

To determine if these receptors are involved in reconsolidating remote object memories (48 h) in PRh, remote SOR parameters were used (Figure 4b) and rats were given an infusion of vehicle or Ro immediately following reactivation with novelty. Post-reactivation infusion of Ro had no effect on memory performance, suggesting these receptors are not necessary for reconsolidating remote object memories (Figure 4d). A paired samples *t*-test demonstrated that choice DRs were not significantly different (*t*(8) = −0.91, *p* = 0.39) when rats were given vehicle infusions or Ro infusions. Both groups displayed intact memory during the choice phase, as paired-sample *t*-tests comparing sample-to-choice DRs were significantly different in both conditions (all *p* values < 0.001).

### 2.5. GluN2A-Containing NMDARs in PRh Are Not Required for Destabilizing or Reconsolidating Remote Object Memories

**Figure 5:**
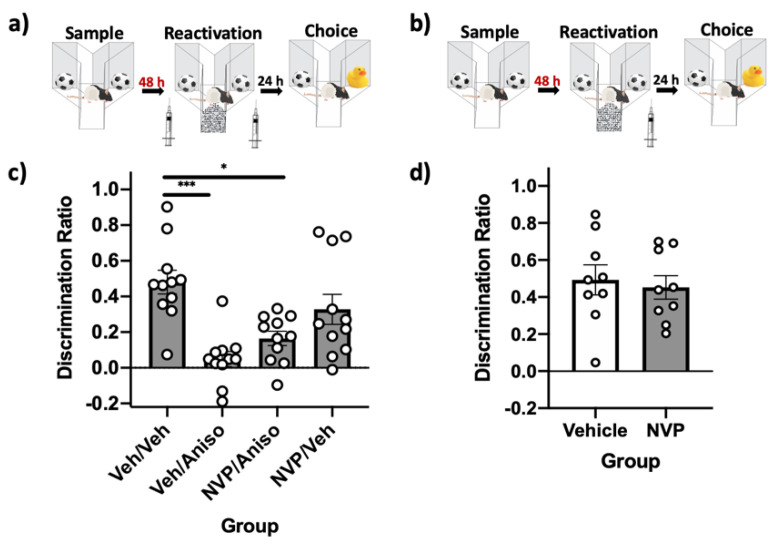
GluN2A-containing NMDARs in PRh are not required for destabilizing or reconsolidating remote object memories. (**a**) Remote SOR parameters, where a floor insert is required at the time of reactivation to destabilize remote object memories. PRh infusions were performed immediately prior to reactivation to target destabilization and prevent the impairing effects of post-reactivation infusion of anisomycin. (**b**) Remote SOR parameters, where only post-reactivation PRh infusions were performed to target reconsolidation. (**c**) Choice-phase DRs demonstrating that both groups receiving post-reactivation anisomycin were significantly impaired compared to the veh–veh condition. The veh–veh and NVP–veh groups had choice DRs that differed from their respective sample DRs, suggesting that only these groups had intact memory. (**d**) Choice-phase DRs demonstrating memory did not differ between groups. Both groups’ choice DRs differed significantly from their respective sample DRs, suggesting memory was intact. Bars are mean DR ± SEM; * p < 0.05, *** *p* < 0.001.

These experiments investigated the involvement of GluN2A-containing NMDARs in PRh in remote (48 h) object memory destabilization (*n* = 11) and reconsolidation (*n* = 9). First, using the remote SOR parameters where a floor insert is placed to serve as contextual novelty(Figure 5a), we tested the involvement of these receptors in memory destabilization. Immediate post-reactivation infusion of the protein synthesis inhibitor anisomycin impaired object memory, demonstrating that these conditions are sufficient to destabilize remote object memories; this impairment persisted despite pre-reactivation infusion of NVP (Figure 5c). This suggests that similar to what was observed under standard conditions, GluN2A-containing NMDARs do not appear to be involved in remote object memory destabilization. A repeated measures one-way ANOVA revealed a significant effect of the drug (*F*(3,30) = 7.92, *p* = 0.00, *η*^2^ = 0.44) on choice DR. Post-hoc analysis determined that performance in the vehicle–anisomycin and NVP–anisomycin conditions was significantly impaired relative to the vehicle–vehicle condition (all *p* values < 0.05). Paired-sample *t*-tests revealed that sample and choice DRs were not significantly different in the vehicle–anisomycin (*p* = 0.09) or NVP–anisomycin (*p* = 0.07) conditions, suggesting impaired object recognition memory. Memory was intact in the vehicle–vehicle and NVP–vehicle conditions, as demonstrated by a significant difference between sample and choice DRs (*t*(10) = −3.19, *p* = 0.01; *t*(10) = −3.54, *p* = 0.005).

There was a significant effect of the drug condition on total exploration in the sample (*F*(3,30) = 5.06, *p* = 0.024, *η*^2^ = 0.34) and reactivation phases (*F*(3,30) = 6.13, *p* = 0.002, *η*^2^ = 0.38). During the sample phase, post-hoc tests demonstrated that there was significantly reduced exploration in the NVP–vehicle group compared to the NVP–anisomycin group (*p* = 0.04). However, this reduction in exploration did not appear to affect performance in the NVP–vehicle condition, as recognition memory was intact in the choice phase. Similarly, at the time of reactivation, exploration was significantly reduced in the NVP–vehicle condition compared to the vehicle–anisomycin (*p* = 0.01) and NVP–anisomycin (*p* = 0.02) groups, but this did not appear to affect choice performance.

To investigate the involvement of these receptors in PRh in reconsolidating remote (48 h) object memories, the remote SOR parameters were used and rats were given an infusion of NVP immediately following reactivation (Figure 5b). Unlike what was observed with the standard SOR parameters, post-reactivation infusion of NVP had no effect on memory performance, suggesting that these receptors are not required for reconsolidating remote object memories (Figure 5d). A paired samples *t*-test demonstrated that there was no significant difference in choice DRs (*t*(8) = −0.42, *p* = 0.69) when rats were infused with NVP or vehicle following reactivation with novelty. Paired-sample *t*-tests comparing sample-to-choice DRs demonstrate that memory was intact in the vehicle (*t*(8) = −6.63, *p* = 0.00) and NVP conditions (*t*(8) = −4.91, *p* = 0.001).

The vehicle condition had significantly lower object exploration during the sample phase (*t*(8) = 2.28, *p* = 0.05); however, this reduced exploration did not appear to affect memory performance, as memory was intact during the choice phase.

### 2.6. GluN2B-Containing NMDARs in PRh Are Not Required for M_1_-Induced Destabilization of Remote Object Memories

**Figure 6:**
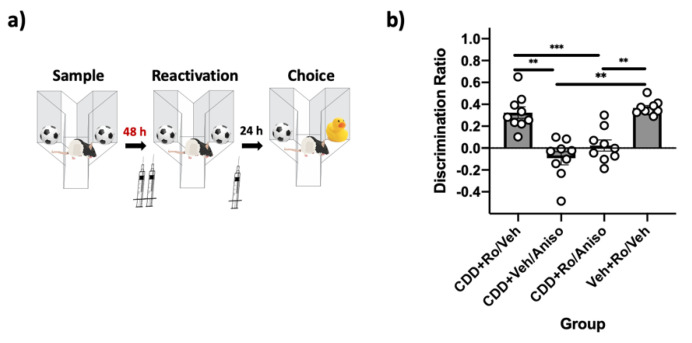
Destabilization of remote memories promoted by M_1_-receptor activation in PRh does not require GluN2B-containing NMDARs. (**a**) Remote SOR parameters were used, but the contextual novelty was withheld during reactivation and instead destabilization was induced by activating M_1_-receptors in PRh prior to reactivation. (**b**) Choice-phase DRs. Groups that received anisomycin following reactivation had significantly impaired memory performance compared to post-reactivation veh conditions, suggesting that CDD infusion prior to reactivation was sufficient to induce destabilization and that co-infusion of Ro-25 did not prevent M_1_-induced destabilization. Bars are mean DR ± SEM; ** *p* < 0.01, *** *p* < 0.001.

Above, we have shown that NMDAR antagonism does not prevent novelty-induced destabilization of remote object memories, an effect we have previously demonstrated to be mAChR-dependent [24]. The purpose of this experiment was to determine if GluN2B-containing NMDARs are required for M_1_-induced remote object memory destabilization (*n* = 9) to further evaluate whether this cholinergic mechanism is independent of NMDAR contributions. The remote SOR parameters were used, but the floor insert was not present during reactivation (Figure 6a). Destabilization was instead induced through activation of M_1_ receptors in PRh by infusing the selective M_1_ receptor agonist CDD-0102A (CDD) prior to reactivation [24], which was confirmed as post-reactivation infusions of anisomycin were shown to impair object memory (Figure 6b). When the GluN2B-containing NMDAR antagonist Ro was co-infused with CDD prior to reactivation, anisomycin given post-reactivation still impaired object memory (Figure 6b). This suggests that destabilization initiated by M_1_ receptor activation does not require GluN2B-containing NMDARs. A repeated measures ANOVA demonstrated a significant effect of the drug (*F*(3,24) = 24.88, *p* = 0.00, *η*^2^ = 0.76) on DR in the choice phase. Post-hoc analysis determined that choice DRs in the CDD+vehicle–anisomycin and CDD+Ro–anisomycin conditions were not significantly different (*p* = 0.50) from one another and both were significantly different from the CDD+Ro–vehicle (*p* values < 0.01) and the vehicle+Ro–vehicle (*p* values < 0.01) conditions. Paired-sample *t*-tests comparing sample-to-choice DRs were not significantly different in the CDD+vehicle–anisomycin (*t*(8) = 1.86, *p* = 0.10) and the CDD+Ro–anisomycin (*t*(8) = −1.34, *p* = 0.22) conditions, suggesting that object memory was impaired. Memory was intact in the other two conditions (*p* values < 0.01).

### 2.7. GluN2A, GluN2B, and GluN1 Subunit Proteins in PRh, and Not HPC, Are Significantly Increased 24 h Following Learning

**Figure 7:**
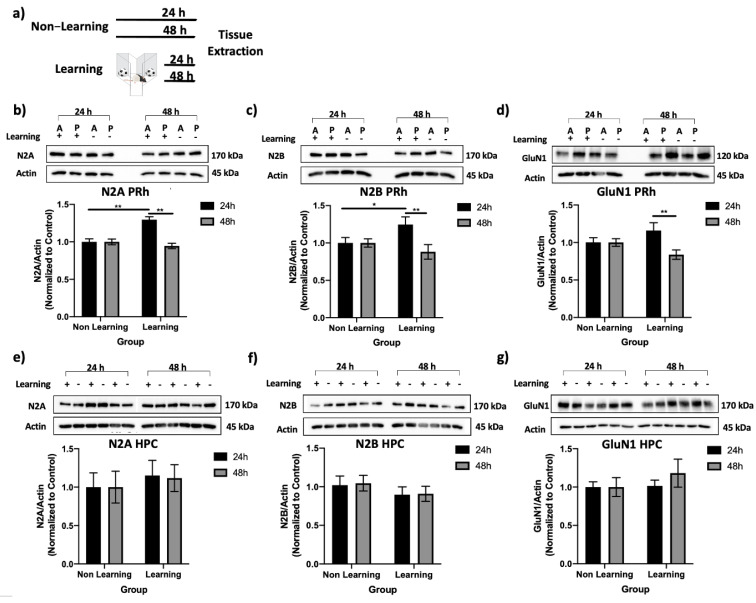
GluN2A, GluN2B, and GluN1 levels are significantly greater 24 h following learning compared to 48 h in PRh but not HPC. (**a**) Schematic of behavioral parameters. Rats in the learning group underwent a series of sample phases, then PRh and HPC tissues were collected either 24 or 48 h later. Non-learning controls remained in home cage. (**b**) Representative blot of GluN2A in anterior (A) and posterior (P) PRh in learning (+) and non-learning (-) groups 24 and 48 h following learning. GluN2A in whole PRh was significantly greater 24 h following learning compared to non-learning controls and 48 h following learning. (**c**) Representative blot of GluN2B in anterior and posterior PRh. GluN2B was significantly increased in whole PRh 24 h following learning compared to non-learning controls and 48 h following learning. (**d**) Representative blot of GluN1 in anterior and posterior PRh. GluN1 in PRh was significantly greater 24 vs 48 h following learning, but was not different from non-learning controls. (**e**) Representative blot of GluN2A in HPC. GluN2A levels did not differ across groups at 24 or 48 h following learning. (**f**) Representative blot of GluN2B in HPC. GluN2B levels did not differ across groups at 24 or 48 h following learning. (**g**) Representative blot of GluN1 in HPC. GluN1 levels did not differ across groups 24 or 48 h following learning. Bars represent mean GluN2 or GluN1 target–actin normalized to control ± SEM; * *p* < 0.05, ** *p* < 0.01.

PRh and hippocampus (HPC) tissue samples were collected 24 or 48 h following object learning in order to assess levels of GluN2A-, GluN2B-, and GluN1 NMDAR subunits related to “standard” and “remote” object memory conditions used in the current study (Figure 7a). Analysis of either GluN2 subunit protein in the anterior and posterior PRh revealed no significant effect of PRh region (N2A, *p* = 0.732; N2B, *p* = 0.198) and no significant interactions of region by learning (N2A, *p* = 0.732; N2B, *p* = 0.198) or delay (N2A, *p* = 0.564; N2B, *p* = 0.429).

When examining levels of GluN2A subunit in whole PRh, however, there was a significant main effect of delay (*F* = 7.02, *p* = 0.01, *η*^2^ = 0.14) and a significant interaction (*F* (1,44) = 7.02, *p* = 0.01, *η*^2^ = 0.01) with no main effect of learning (*F* = 3.34, *p* = 0.07, *η*^2^ = 0.07). GluN2A was significantly increased 24 (*p* = 0.003) but not 48 h *(p* = 0.564) following learning compared to non-learning controls (Figure 7b). Levels of GluN2A were also significantly greater 24 h following learning compared to 48 h following learning (*t*(22) = 2.90, *p* = 0.008).

Similarly, when examining levels of GluN2B subunit in whole PRh, there was a significant interaction (*F*(1,44) = 4.67, *p* = 0.036, *η*^2^ = 0.10), with a significant effect of delay (*F*(1,44) = 4.67, *p* = 0.036, *η*^2^ = 0.10) and no main effect of learning (*F*(1,44) = 0.57, *p* = 0.453, *η*^2^ = 0.013). GluN2B was significantly increased 24 (*p* = 0.045) but not 48 h (*p* = 0.327) following learning compared to non-learning controls, with levels of GluN2B significantly greater at 24 compared to 48 h in the learning group (*t*(22) = 2.58, *p* = 0.017) (Figure 7c).

Levels of GluN1 subunit in the entire PRh were also examined to determine whether the changes following learning were selective to the GluN2 subtypes. There was a significant interaction (*F*(1,40) = 4.73, *p* = 0.036, *η*^2^ = 0.11), with a significant main effect of delay (*F*(1,40) = 4.73, *p* = 0.036, *η*^2^ = 0.11) but not learning (*F*(1,40) = 0.001, *p* = 0.982, *η*^2^ = 0.00). Levels of GluN1 24 h following learning did not differ significantly compared to non-learning controls (*p* = 0.136); however, within the learning group, GluN1 was significantly greater 24 h following learning compared to 48 h (*t*(20) = 2.42, *p* = 0.025) (Figure 7d).

Levels of GluN2A (Figure 7e), GluN2B (Figure 7f), and GluN1 (Figure 7g) were not changed 24 h or 48 h following learning in the HPC, as there were no significant main effects of delay (N2A, *p* = 0.93; N2B, *p* = 0.86; GluN1, *p* = 0.50), group (N2A, *p* = 0.48, N2B, *p* = 0.22; GluN1, *p* = 0.42) or the interaction term (N2A, *p* = 0.93; N2B, *p* = 0.94; GluN1, *p* = 0.50). This suggests that changes in levels of NMDA receptor subunit proteins in the current study were relatively selective to PRh.

Differences in levels of GluN2A, GluN2B, and GluN1 subunits at 24 and 48 h following learning could not be accounted for by differences in object exploration, as total object exploration did not differ between rats sacrificed at 24 and 48 h (*t*(22) = −1.52, *p* = 0.142).

## 3. Discussion

In the present study, we identified a role for glutamate in destabilizing and reconsolidating relatively recent object memories in PRh when acting on GluN2B- and GluN2A-containing NMDARs, respectively. However, the dissociable involvement of GluN2B- and GluN2A-containing NMDARs in object memory destabilization and reconsolidation appears to be temporally limited, such that when object memories are older and resistant to destabilization, neither receptor subtype is necessary for novelty-induced destabilization or reconsolidation. Consistent with these findings, we demonstrate that GluN2B and GluN2A subunit proteins in PRh are increased 24 h following learning but return to non-learning control levels after 48 h. Changes in levels of GluN2 subunit composition following learning were found to be selective to PRh, suggesting that these changes are the result of object learning specifically. In contrast, and consistent with our past work implicating mAChRs in object memory destabilization and updating [24,35,37], we demonstrated that M_1_ mAChR activation can override this boundary condition and does so independently of GluN2B-containing NMDARs. This is the first study to differentiate the roles of these two important neuromodulators of memory destabilization, and in doing so it complements previous findings regarding the involvement of NMDARs, while emphasizing the important role of ACh for destabilizing resistant memories.

To our knowledge, this is the first study to demonstrate the dissociable roles for GluN2B- and GluN2A-containing NMDARs found to underlie auditory fear memory destabilization and reconsolidation [15], respectively, in another brain region for a different form of memory. In the present study, inhibition of GluN2B-containing NMDARs in PRh prevented destabilization of relatively recent object memories, similar to what has been observed in previous work by our group investigating the role of M_1_ receptors [24]. Together these results suggest that both glutamate and ACh in PRh are required for destabilizing relatively recent object memories in the absence of salient novelty or prediction error. Activation of GluN2B-containing NMDARs and M_1_-receptors can lead to increases in intracellular calcium, which can in turn regulate the UPS through calcium/calmodulin-dependent protein kinase II (CaMKII) activation [20,22,24]. Activation of both receptor types might be required to produce a sufficiently large calcium signal under such conditions to activate the UPS to carry out synaptic destabilization; however, further research would be required to investigate this possible interaction.

We further extended the findings of Milton et al. (2013) [15] by demonstrating that the respective contributions of GluN2B- and GluN2A-containing NMDARs are negated when boundary conditions are in place and object memories are resistant to destabilization. The reduced involvement of GluN2B-containing NMDARs in memory destabilization has been implied in previous research demonstrating reductions in GluN2B-containing NMDARs after strong fear conditioning protocols [31,32]; however, in the present study, we provide behavioral evidence for the reduced functionality of these receptors for destabilizing remote, resistant memories.

Based on previous work demonstrating that reductions in GluN2B-containing NMDARs are important for establishing boundary conditions to reconsolidation for other types of learning and memory [31,32], we predicted that a similar mechanism underlies remote object memory resistance to destabilization in PRh. While we observed a similar trend, whereby older (48 h) object memories that are resistant to destabilization were associated with lower levels of GluN2B subunit protein than relatively recent (24 h) memories, comparisons to non-learning controls suggest that GluN2B-containing NMDARs are increased 24 h following learning and return to baseline levels at 48 h. Thus, a more accurate interpretation of the present findings may be that glutamate is temporarily involved in object memory destabilization due to an increase in GluN2B-containing NMDARs following learning; as the time after acquisition increases and these receptors return to baseline levels, there is a corresponding reduction in the role of glutamate in memory destabilization. This may in turn reduce the likelihood that the older memory will destabilize, but this was not directly assessed in the current study.

Previous studies demonstrate that the reduction in GluN2-containing receptors in the BLA underlying strong fear memories is selective to the N2B subtype, with N2A levels staying consistent or even slightly increasing following stronger training protocols, with no changes in GluN1 [31,32]. Interestingly, here we found a similar pattern for both GluN2B and GluN2A subunit proteins in PRh, with levels of both increased at 24 h compared to baseline and 48 h following learning. While levels of GluN1 were not significantly elevated following learning, GluN1 was increased 24 h following learning compared to 48 h. Given the composition of NMDA receptors, where each GluN1 subunit is accompanied by at least one GluN2 subunit, complementary increases in GluN1 could be expected with overall increases in GluN2 subunits following learning. The differences in the findings of this study and others looking at fear memories [31,32] could be the result of the different forms of memory being studied, differences in underlying circuitry of older memories compared to strong memories, as well as differences in the memory storage mechanisms in the BLA and PRh. However, decreased levels of N2A and N2B have also been observed in the BLA 48 h following auditory fear conditioning training with repeated tone–foot shock pairings [38]; that is, there were reduced NMDAR-mediated excitatory post-synaptic currents (EPSC); diminished effects of GluN2B inhibition on EPSCs; and reduced expression of GluN2A, GluN2B, and phosphorylated GluN1 in the BLA 48 h following learning compared to unpaired controls [38]. This study, along with others demonstrating a temporary role for NMDA receptors during initial memory formation [39,40,41], suggests that delayed NMDA receptor downregulation following learning may serve as a mechanism to protect memories from potential disruption following reactivation-induced destabilization [33,38]. Furthermore, the trending increase in GluN1 24 h following learning in PRh observed in this study warrants future investigations to determine if GluN1 has a functional role in memory destabilization and reconsolidation processes. Glycine binding at GluN1 subunits importantly regulates glutamate’s ability to activate NMDA receptors [18] and perhaps plays a role in memory destabilization and reconsolidation by modulating glutamate activation of NMDA receptors.

We further demonstrated that changes in levels of NMDA receptor subunits following object learning may be selective to PRh, as no differences in levels of GluN2A, GluN2B, or GluN1 were found 24 or 48 h following learning in HPC. These findings are consistent with those demonstrating a critical role for PRh but not HPC in object memory encoding, storage, retrieval, and reconsolidation [24,42,43,44,45,46,47]. The selective changes in NMDA receptor subunit composition observed in the present study in PRh but not HPC critically demonstrate that these changes are likely a result of object learning specifically and support the hypothesis that they occur in NMDA receptors associated with object memory.

Despite the reduced involvement of PRh NMDARs in remote object memory reconsolidation, the present findings demonstrate, as in past studies [24,35], that destabilization could nonetheless be initiated through pharmacological activation of M_1_ mAChRs. Previous work by our group has highlighted the mechanism by which M_1_ mAChR activation in PRh could lead to memory destabilization by ultimately activating the UPS [24]. This putative pathway appears to regulate novelty-induced destabilization of remote and strongly encoded object memories, suggesting a special role for ACh in overriding boundary conditions. Critically, activation of this M_1_ pathway in PRh can produce destabilization even in the absence of explicit novelty, and here we demonstrated that this effect occurs while GluN2B-containing NMDARs are antagonized. Thus, the mechanistic effects we previously reported for M_1_ mAChRs in object memory destabilization appear to be independent of any contribution from GluN2B-containing NMDARs, despite the widely demonstrated role for the latter in destabilization of various types of memory in the absence of boundary conditions.

The present findings, therefore, expand our understanding of the neurochemical basis of memory destabilization and reconsolidation in multiple ways. First, we demonstrated that the previously reported double dissociation between the functions of GluN2A- and GluN2B-containing NMDARs in fear memory reconsolidation and destabilization, respectively, also applies to relatively recent long-term object memories in PRh. Second, however, we showed that neither of these NMDAR subtypes is necessary for reconsolidation of relatively remote object memories. Third, this transient role for NMDARs relates to apparent fluctuations in subunit protein levels within PRh, with both significantly increased 24 after learning but returning to baseline by 48h; this is similar to reports of GluN2 subunit protein changes in other brain areas following training protocols that induce boundary conditions on reconsolidation. Finally, in contrast to the transient role for NMDARs, M_1_ mAChRs are involved in destabilization of both recent and remote long-term object memories. 

In summary, the current results, along with the broader literature, imply dueling but complementary roles for NMDARs and mAChRs in memory destabilization; these roles likely relate to the complexity and dynamic nature of long-term memory storage and the importance of its behavioral influence within constantly changing environments. Fluctuations in NMDAR subtypes during the hours and days following learning might represent a mechanism to protect recently acquired long-term memories from subtle, unnecessary changes that could compromise the accuracy of recently stored information. Conversely, ACh acting in mAChRs might play a specialized role in facilitating significant adaptive updating of established memory networks throughout the lifespan. Thus, the present findings could be highly relevant to understanding the nature of cognitive and behavioral (in)flexibility in aging and dementia. Moreover, should this function of ACh be generalizable to other types of memory, the M_1_ mAChR could prove to be a promising target for initiating destabilization of resistant memories associated with conditions such as post-traumatic stress disorder, phobias, and addiction, such that these memories can be modified in therapeutic settings.

## 4. Materials and Methods

### 4.1. Subjects

The subjects were 80 adult male Long–Evans rats (Charles River, QC, Canada), weighing 275–350 g at the onset of testing. All rats were pair-housed in a colony room set on a 12 h reverse light–dark cycle (lights off 8:30–20:30 h). Behavioral testing was conducted during the dark phase. Rats were on restricted feed to encourage exploration during testing; 20 g of rodent chow (Highland Rat Chow) was provided to each cage following behavioral testing to maintain 85–95% free-feed body weight. Water was available ad libitum in home cages.

Cannulated rats were housed in individually ventilated GR1800 Double Decker cages (Techniplast). The cages are made of H-TEMPT polysulfone plastic with 1800 cm^2^ floor area, including the second level, with a total height of 38 cm. Non-cannulated rats were housed in opaque plastic cages with the dimensions 48 × 26 × 20 cm. All cages contained Bed-o-Cob bedding (Harlan Laboratories, Inc., Mississauga, ON, Canada), a brown paper towel, Crink-l’Nest™ (The Andersons, Inc., Maumee, OU, USA), and a 14-cm-long white paper cup 12 cm in diameter. 

### 4.2. Surgical Procedures

Rats in intracranial experiments were implanted bilaterally with 22-gauge indwelling guide cannulas (Plastics1; HRS Scientific, Anjou, QC, Canada) targeting PRh. Prior to surgery, rats were deeply anesthetized using isoflurane (Benson Medical Industries, Markham, ON, Canada) and given an injection of Medicam (5 mg/kg, subcutaneous) and Baytril (50 mg/kg, intramuscular). Using non-puncture ear bars, with an incisor bar set to −3.3 mm, rats were secured to the stereotaxic frame (Kopf Instruments, Tujunga, CA, USA). The scalp and periosteum were cut and retracted to expose the skull. Holes were drilled into the skull to allow guide cannulas to be implanted according to the following coordinates: anteroposterior −5.5 mm, lateral ±6.6 mm, dorsoventral −7 mm. All coordinates were measured relative to bregma [48]. Cannulas were secured to the skull with four jeweler screws and dental acrylic. Dummy cannulas measuring 0.36 mm in diameter and cut to extend 1.1 mm beyond the guide cannulas were placed into the guide cannulas and removed only during testing. Following surgery, the incision was sutured and rats were placed in an empty cage on heat to recover for 1 hour. All rats were given one week to recover in their home cages prior to behavioral testing.

### 4.3. Intracranial Drugs

The protein synthesis inhibitor anisomycin (Sigma-Aldrich, Oakville, ON, Canada) was dissolved in 1 N hydrochloric acid (HCl) and neutralized with sodium hydroxide (NaOH) to a pH of 7.4. Physiological saline (0.9%) was used to adjust the final concentration to 100 µg/µL [24,35,46]. The selective GluN2A-containing NMDAR antagonist NVP-AAM077 tetrasodium hydrate (NVP; Sigma-Aldrich, Oakville, ON, Canada) was dissolved in PBS to a final concentration of 5 µg/µL [15]. The selective GluN2B-containing NMDAR antagonist Ro 25-6981 (Ro; Sigma-Aldrich, Oakville, ON, Canada) was dissolved in 40% DMSO and phosphate-buffered saline (PBS) to a final concentration of 2 µg/µL. The M_1_ receptor agonist CDD-0102A (CDD; generously donated by Dr. William Messer, University of Toledo) was dissolved in 0.9% physiological saline to a concentration of 1 µg/µL [24,49]. The vehicle control was selected according to the primary solvent for each corresponding drug.

### 4.4. Microinfusion Procedure

Microinfusions took place in a preparation room that was separate from the behavioral testing room. Rats were either given two microinfusions, one prior to reactivation and one immediately following, when testing object memory destabilization, or one microinfusion immediately following reactivation when testing object memory reconsolidation.

At the time of infusion, rats were gently restrained and the dummy cannulas were removed to allow for insertion of a 28 gauge infusion cannula. The infusion cannulas were inserted into propylene tubing attached to two 1 µL Hamilton syringes to allow for simultaneous bilateral infusions. Syringes were fastened to a Harvard Apparatus (Hilliston, MA, USA) precision syringe pump set to deliver infusions of 1.0 µL over 2 min. Infusers were left in place for 1.5 min following infusions to allow for diffusion of the infusate. Infusers were then removed and dummy cannulas were reinserted.

### 4.5. Histology

Following each behavioral experiment, rats were anesthetized with 1.0 mL intra peritoneal (I.P.) Euthansol (340 mb/mL; MERK, Intervet Canada Corp, Kirkland, QC, Canada) and transcardially perfused with PBS followed by 4% neutral buffered formalin. Brains were extracted and post-fixed in 4% formalin at 4 °C for at least 24 h before being transferred to 20% sucrose in PBS. Once the brains had sunk, they were mounted in a cryostat and sliced into coronal sections (55 µm) through PRh. Every third slice was mounted on a gelatin-coated glass slide to be stained with cresyl violet. Slides were examined under a light microscope to verify cannula placements.

### 4.6. General Procedure

All experiments used a modified SOR task conducted in a Y-shaped apparatus, as previously described [47]. Object exploration in all phases of the experiment was scored manually using a custom computer program and recorded on a JVC camera mounted above the apparatus. The experiments followed the same general procedure as those previously published [16,24]. Briefly, all rats were given two habituation sessions on two consecutive days. Rats were given a mock infusion (no infusate) prior to being placed in an empty apparatus that they were allowed to explore freely for 3 min. Each trial consisted of three phases: sample, reactivation, and choice. During the sample phase, rats were allowed to explore a pair of identical objects, one object placed in each arm of the apparatus, for 3 min or 30 s of recorded object exploration, whichever came first. At the time of reactivation, rats were re-presented the same objects from the sample phase and allowed to explore for 2 min or 10 s of recorded object exploration, whichever came first. During the choice phase, rats were presented with the “familiar” sample object in one arm and a novel object in the other and allowed to explore for 2 min. The “standard” SOR procedure utilized a 24 h delay between the sample and reactivation phase. Previous studies demonstrated that under these conditions object memories will readily destabilize when the objects are briefly re-presented during the reactivation phase [16,24,35]. The “remote” SOR procedure utilized a 48 h delay between the sample and reactivation phase, which has previously been shown to promote the development of a memory trace that resists destabilization unless the reactivation context is manipulated to contain novel information [16,24,35]. In accordance with previous studies, a cardboard floor insert covered in various materials (e.g., felt, rubberized drawer liner) served as the novel contextual change during the reactivation [16,24,35]. The arm containing the novel object, the familiar and novel objects, and the texture of the floor insert were all counterbalanced between trials. All behavioral experiments were conducted as within subjects. A new pair of objects was used for each trial such that no rat saw the same object on more than one trial.

### 4.7. Protein Isolation and Western Blots

Forty-eight rats were habituated to the apparatus (as described in Section 4.6) and assigned to one of four conditions (*n* = 12/group). Both the 24 and 48 h learning groups were given 30 separate sample phases, with a new, distinct object pair presented during each sample, in order to establish 30 object representations. We used this procedure in order to ensure a sufficient signal to detect differences in synaptic proteins underlying object representations. Previous work from our group has demonstrated the efficacy of this procedure for detecting differences in *c-fos* activity in PRh following object exploration [50]. The non-learning groups remained in the home cage until tissue collection, which was timed to match to that of either the 24 or 48 h learning group to control for time of tissue extraction. Either 24 or 48 h following learning, rats were briefly exposed to CO_2_ and brains were rapidly extracted. The entire bilateral PRh was excised and separated evenly into anterior and posterior halves, placed in a tube, and frozen on dry ice. Samples were maintained at −80 °C until processed.

PRh and HPC synapses were isolated by homogenizing samples using Syn-PER™ Synaptic Protein Extraction Reagent (Thermo Fisher Scientific). A protease inhibitor tablet (Pierce) was added to the Syn-PER™ reagent and samples were homogenized and centrifuged at 1200× *g* for 10 min at 4 °C. The pellet was discarded, and the supernatant was centrifuged at 15,000× *g* for 20 min at 4 °C. The synaptosomal pellet was re-suspended in Syn-PER™ reagent and protein concentrations were determined using a Bradford assay.

Samples (20 µg total protein/well) were loaded onto 8% sodium dodecyl sulfate polyacrylamide (SDS-PAGE) gels and electrophoresis was performed using a Mini-PROTEAN Tetra cell system (Bio-Rad, Mississauga, ON, Canada). Gels were run for 2 h before proteins were transferred onto a 0.45 mM nitrocellulose (Bio-Rad) membrane using the Trans-Blot SD Turbo transfer apparatus (Bio-Rad) at 25 V (constant voltage) for 30 min. Blots were then rinsed briefly in tris-buffered saline with 0.1% Tween-20 (TBS-T) before being blocked in either 3% non-fat milk or 5% bovine serum albumin (BSA; Roche, Indianapolis, Indiana, USA) for 1 h. Blots were rinsed twice in TBS-T and left to incubate in primary antibodies overnight at 4 °C. Conditions for each antibody were as follows: GluN2A (Millipore Sigma, Milwaukee, WI, USA, 07-632; blocking: 3% milk in TBS-T; primary: 1:1000 in 3% milk in TBS-T; secondary: anti-rabbit 1:2500 in 3% milk in TBS-T); GluN2B (Sigma-Aldrich, Oakville, ON, Canada, 06-600; blocking: 3% milk in PBS; primary: 1:1000 in 3% milk in PBS; secondary: anti-rabbit 1: 2500 in 3% milk in PBS); B-Actin (Cell Signaling Technology, Whitby, ON, Canada, 8H10D10; blocking: 5% BSA in 0.1% TBS-T; primary: 1:5000 in 5% BSA in 0.1% TBS-T; secondary: anti-mouse 1:2500 in 5% BSA in 0.1% TBS-T); GluN1 (Cell Signaling Technology; 5704S; blocking: 3% BSA in 0.1% TBS-T; primary 1:1000 in 3% BSA in 0.1% TBS-T; secondary: anti-mouse 1:2500 in 3% BSA in 0.1% TBS-T). Blots were rinsed twice in TBS-T and incubated for 1 h at room temperature with horseradish peroxidase (HRP)-conjugated goat anti-mouse or goat anti-rabbit IgG (1:2500; Cell Signaling) in milk or BSA. Blots were rinsed twice for 5 min and twice for 10 min in TBS-T before target proteins were visualized with Luminata Forte HRP substrate (Millipore-Sigma, Oakville, ON, Canada) on a ChemiDoc MP imaging system (Bio-Rad). Densitometry was performed using mage Lab v4.1 software (Bio-Rad).

### 4.8. Object Recognition Data Analysis

There were two variables analyzed for each behavioral testing phase; the sample and reactivation phases were analyzed for total object exploration and the choice phase was analyzed for total object exploration and DR. All DR and exploratory means for each experiment can be found in the Supplementary Material (Tables S1–S3). The DR is a representation of the proportional difference between the amount of time spent exploring the novel object compared to that of the familiar object (Equation (1)):

(1) $$\mathrm{DR}\;=\;\frac{\left(1\;\min\;\mathrm{novel}\;\mathrm{exploration}\;\mathrm{time}\;–\;1\;\min\;\mathrm{familiar}\;\mathrm{exploration}\;\mathrm{time}\right)\;}{\left(\mathrm{total}\;\mathrm{novel}\;\mathrm{exploration}\;\mathrm{time}\;+\;\mathrm{total}\;\mathrm{familiar}\;\mathrm{exploration}\;\mathrm{time}\right)}$$

A mock DR was also calculated for the sample phase (Equation (2)):

(2) $$\mathrm{Mock}\;\mathrm{DR}\;=\;\frac{\left(\mathrm{exploration}\;\mathrm{in}\;\mathrm{arm}\;\mathrm{containing}\;\mathrm{novel}\;\mathrm{object}\;\mathrm{at}\;\mathrm{choice}\;–\;\mathrm{exploration}\;\mathrm{in}\;\mathrm{the}\;\mathrm{arm}\;\mathrm{containing}\;\mathrm{familiar}\;\mathrm{object}\;\mathrm{at}\;\mathrm{choice}\right)}{\left(\mathrm{total}\;\mathrm{time}\;\mathrm{exploration}\;\mathrm{in}\;\mathrm{novel}\;\mathrm{and}\;\mathrm{familiar}\;\mathrm{arms}\right)}$$

to rule out exploration preferences in the Y-apparatus. Total object exploration was utilized as a control measure to rule out drug effects on object exploration. Choice DRs and total exploration in each phase were analyzed using repeated measures one-way ANOVA and independent or paired-sample *t*-tests in the case of planned comparisons or for post-hoc tests to probe significant main effects. In addition, paired-sample *t*-tests were used to compare sample and choice DRs in each condition of an experiment, as a DR of 0 is expected in the sample phase when the identical objects are equally novel. A significant difference between sample and choice DR indicates discrimination between the familiar and novel objects in the choice phase and is interpreted as intact memory. Outliers greater or less than two standard deviations from the mean were removed and replaced with the group mean. All statistical analyses were conducted using SPSS version 26 (IBM Corp., Armonk, NY, USA), with a significance value of α = 0.05. For post-hoc *t*-tests exploring significant main effects of the drug condition, significance values were adjusted using the Bonferroni correction.

### 4.9. Western Blot Data Analysis

Values obtained from a single rat’s anterior and posterior PRh were analyzed both separately as well as pooled together to provide a measure of protein levels in the whole PRh for each rat. Western blot densitometric quantitative data were analyzed using 2 × 2 (delay, learning) ANOVA or 2 × 2 × 2 (delay, learning, PRh region) mixed factorial ANOVA, and post-hoc tests (Bonferroni-corrected) were performed in the case of a significant interaction to probe the effect of learning within each delay. Comparisons of GluN2 protein subtypes in whole PRh in the 24 and 48 h groups were analyzed in an a priori manner using a two-tailed independent samples *t*-test. To control for variability in protein loading, target protein densities were corrected to actin and compared to non-learning controls.

Object exploration across all sample phases was averaged for each rat and mean total exploration between the 24 and 48 h learning groups was compared using a two-tailed independent samples *t*-test to assess differences in overall exploration. All statistical analysis for Western blot and exploration data were performed with SPSS version 26 (IBM Corp., Armonk, NY, USA), with a significance value of α = 0.05.

## Data Availability

The behavioural data presented in the study are available in the supplementary material (Tables S1–S3). Western blot data are available on request from the corresponding author. These data are not publicly available for privacy reasons.

## Supplementary Materials

Supplementary tables containing means and SEMs for control measures calculated for behavioral experiments can be found at https://www.mdpi.com/1422-0067/22/1/67/s1.

## Abbreviations

ACh	Acetylcholine
ANOVA	Analysis of variance
BLA	Basolateral amygdala
BSA	Bovine serum albumin
CaMKII	Calcium/calmodulin-dependent protein kinase II
CDD	CDD-0102A
DR	Discrimination ratio
EPSC	Excitatory post-synaptic currents
HCl	Hydrochloric acid
HPC	Hippocampus
HRP	Horseradish peroxidase
mAChR	Muscarinic acetylcholine receptor
NaOH	Sodium hydroxide
NMDA	N-methyl-D-aspartate
NVP	NVP-AAM 077
PBS	Phosphate-buffered saline
PRh	Perirhinal cortex
Ro	Ro 25-6981
SDS-PAGE	Sodium dodecyl sulfate polyacrylamide
SOR	Spontaneous object recognition
TBS-T	Tris-buffered saline-0.1% Tween-20
UPS	Ubiquitin proteasome system
