# Supplementary material for: Fluctuating NMDA Receptor Subunit Levels in Perirhinal Cortex Relate to Their Dynamic Roles in Object Memory Destabilization and Reconsolidation
Source: Int J Mol Sci. 2020 Dec 23;22(1):67. doi: 10.3390/ijms22010067 (PMC7793502; doi:10.3390/ijms22010067)
Supplement: Supplementary file 1 [file ijms-22-00067-s001.pdf]

Table S1. Control measures for data collected during the sample phase. Expressed as mean ± standard error

Exp.	*Drug Group*	*Object exploration*	*Discrimination Ratio*
2.2	Veh/Veh	14.59 ± 1.12	-0.09 ± 0.06
	Veh/Ansio	24.0 ± 1.39	0.09 ± 0.06
	Ro/Aniso	20.77 ± 1.38	-0.04 ± 0.06
	Ro/Veh	12.75 ± 1.01	-0.05 ± 0.07
	Veh	16.26 ± 2.09	0.00 ± 0.06
	Ro	16.32 ± 1.95	0.11 ± 0.07
2.3	Veh/Veh	12.82 ± 0.80	-0.14 ± 0.03
	Veh/Aniso	16.68 ± 1.29	-0.003 ± 0.02
	NVP/Aniso	15.90± 2.46	-0.04 ± 0.04
	NVP/Veh	14.16 ± 0.93	0.05 ± 0.07
	Veh	9.93 ± 0.88	0.02 ± 0.05
	NVP	14.18 ± 1.21	0.02 ± 0.03
2.4	Veh/Veh	17.75 ± 2.09	0.03 ± 0.08
	Veh/Aniso	21.43 ± 0.79	0.04 ± 0.07
	Ro/Aniso	18.74 ± 2.11	-0.001 ± 0.06

	Ro/Veh	15.54 ± 1.78	0.01 ± 0.04
	Veh	14.62 ± 1.39	-0.03 ± 0.05
	Ro	14.38 ± 2.23	0.004 ± 0.06
2.5	Veh/Veh	11.59 ± 0.56	0.13 ± 0.06
	Veh/Aniso	11.48 ± 1.14	-0.09 ± 0.06
	NVP/Aniso	16.69 ± 2.25	0.001 ± 0.06
	NVP/Veh	9.63 ± 0.86	-0.12 ± 0.08
	Veh	10.34 ± 1.20	0.06 ± 0.07
	NVP	14.83 ± 1.18	-0.01 ± 0.06
2.6	CDD+Ro/Veh	16.09 ± 2.37	-0.10 ± 0.06
	CDD+Veh/Aniso	10.86 ± 1.39	0.08 ± 0.04
	CDD+Ro/Aniso	14.30 ± 1.82	-0.05 ± 0.05
	Veh+Ro/Veh	14.13 ± 1.58	-0.07 ± 0.07

Table S2. Control measures for data collected during the reactivation phase. Expressed as mean ± standard error.

Exp.	*Drug Group*	*Object exploration*
2.2	Veh/Veh	8.46 ± 0.49
	Veh/Ansio	8.42 ± 0.42
	Ro/Aniso	7.90 ± 0.58
	Ro/Veh	9.20 ± 0.37
	Veh	8.45 ± 0.60
	Ro	8.03 ± 0.58
2.3	Veh/Veh	9.67 ± 0.26
	Veh/Aniso	10.04 ± 0.01
	NVP/Aniso	9.60 ± 0.25
	NVP/Veh	9.08 ± 0.38
	Veh	9.47 ± 0.41
	NVP	8.93 ± 0.42
2.4	Veh/Veh	9.74 ± 0.12
	Veh/Aniso	7.11 ± 0.72
	Ro/Aniso	8.90 ± 0.42

	Ro/Veh	9.00 ± 0.38
	Veh	9.04 ± 0.53
	Ro	7.61 ± 0.78
2.5	Veh/Veh	4.76 ± 0.75
	Veh/Aniso	5.93 ± 0.63
	NVP/Aniso	6.68 ± 0.90
	NVP/Veh	3.13 ± 0.33
	Veh	8.04 ± 0.85
	NVP	7.60 ± 0.52
2.6	CDD+Ro/Veh	7.36 ± 0.73
	CDD+Veh/Aniso	6.15 ± 0.91
	CDD+Ro/Aniso	6.31 ± 0.83
	Veh+Ro/Veh	8.20 ± 0.70

Table S3. Control measures for data collected during the choice phase. Expressed as mean ± standard error.

Exp.	*Drug Group*	*Object exploration*	*Discrimination Ratio*
2.2	Veh/Veh	14.91 ± 1.39	0.43 ± 0.06
	Veh/Ansio	16.60 ± 1.32	0.11 ± 0.03
	Ro/Aniso	15.07 ± 1.31	0.53 ± 0.05
	Ro/Veh	15.88± 0.97	0.39 ± 0.08
	Veh	10.48 ± 1.05	0.62 ± 0.06
	Ro	11.44 ± 1.63	0.64 ± 0.04
2.3	Veh/Veh	11.44 ± 1.42	0.46 ± 0.07
	Veh/Aniso	14.11 ± 1.27	-0.05 ± 0.05
	NVP/Aniso	13.58 ± 1.42	0.09 ± 0.03
	NVP/Veh	14.36 ± 1.69	0.07 ± 0.03
	Veh	9.96 ± 0.70	0.53 ± 0.03
	NVP	13.50 ± 1.27	0.06 ± 0.05
2.4	Veh/Veh	13.15 ± 1.30	0.39 ± 0.07
	Veh/Aniso	14.07 ± 1.31	0.04 ± 0.06
	Ro/Aniso	14.14 ± 1.42	-0.05 ± 0.07

	Ro/Veh	11.29 ± 1.44	0.45 ± 0.07
	Veh	15.37 ± 2.62	0.40 ± 0.03
	Ro	16.19 ± 1.13	0.49 ± 0.09
2.5	Veh/Veh	12.33 ± 0.95	0.48 ± 0.07
	Veh/Aniso	13.27 ± 1.80	0.05 ± 0.04
	NVP/Aniso	12.05 ± 1.34	0.16 ± 0.04
	NVP/Veh	13.68 ± 1.41	0.33 ± 0.08
	Veh	10.27 ± 1.33	0.49 ± 0.08
	NVP	11.37 ± 1.51	0.45 ± 0.06
2.6	CDD+Ro/Veh	16.97 ± 2.90	0.32 ± 0.05
	CDD+Veh/Aniso	13.15 ± 0.5	-0.09 ± 0.05
	CDD+Ro/Aniso	16.53 ± 1.62	0.02 ± 0.05
	Veh+Ro/Veh	17.01 ± 1.29	0.34 ± 0.02
